# A dosimetric comparison of helical tomotherapy treatment delivery with real-time adaption and no motion correction

**DOI:** 10.1016/j.phro.2025.100741

**Published:** 2025-03-05

**Authors:** Jonathan Hindmarsh, Scott Crowe, Julia Johnson, Chandrima Sengupta, Jemma Walsh, Sonja Dieterich, Jeremy Booth, Paul Keall

**Affiliations:** a Image X Institute, Faculty of Medicine and Health, University of Sydney, Eveleigh, NSW, Australia; b Cancer Care Services, Royal Brisbane and Women’s Hospital, Herston, QLD, Australia; c Department of Radiation Oncology, UC Davis Medical Center, Sacramento, CA, USA; d Northern Sydney Cancer Centre, Royal North Shore Hospital, St Leonards, NSW, Australia; e Institute of Medical Physics, School of Physics, University of Sydney, Camperdown, NSW, Australia

**Keywords:** MLC tracking, Organ motion, Real-time adaptive radiotherapy, Helical tomotherapy

## Abstract

This study assesses the ability of a helical tomotherapy system equipped with kV imaging and optical surface guidance to adapt to motion traces in real-time. To assess the delivery accuracy with motion, a unified testing framework was used. The average 2 %/2 mm γ-fail rates across all lung traces were 0.1 % for motion adapted and 17.4 % for no motion correction. Average 2 %/2 mm γ-fail rates across all prostate traces were 0.4 % for motion adapted and 12.2 % for no motion correction. Real-time motion adaption was shown to improve the accuracy of dose delivered to a moving phantom compared with no motion adaption.

**MeSH Keywords:** Radiotherapy, image-guided; Radiation therapy, targeted.

## Highlights

• Testing a helical tomotherapy system equipped with kV imaging and surface guidance.
• A unified testing framework compared real-time adaption versus no motion correction.
• Average reduction in 2 %/2 mm γ-fail rate was 17.3 % across 5 lung traces.
• Average reduction in 2 %/2 mm γ-fail rate was 11.8 % across 3 prostate traces.
• Helical tomotherapy system performed comparably to other real-time adaptive methods.

## Graphical abstract

**Figure:**
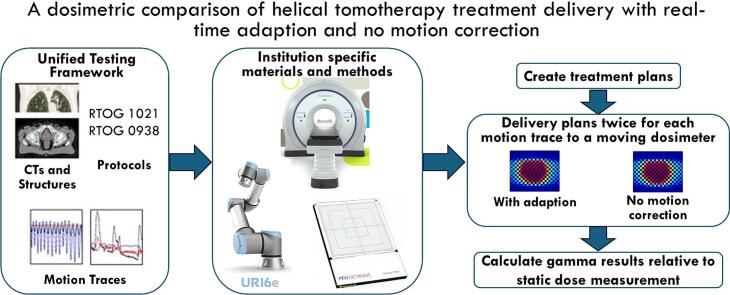

## 1 Introduction

Motion of targets is a challenge to accurate radiation therapy treatment delivery. There is nearly continuous movement of the internal organs in a patient caused by respiration, digestion, circulation and involuntary and voluntary musculo-skeletal movements [[1], [2], [3]]. The presence of motion during a radiotherapy treatment delivery can lead to underdosing of the target and/or overdosing of the normal tissue [[4], [5], [6], [7]]. With the trend to using fewer fractions, motion will have an even greater impact on delivery accuracy in the future [[8], [9], [10]].

Consequently, there has been much research into methods of compensating for and/or managing motion to reduce its impact on treatment accuracy [11,12]. The primary methods of compensating for motion have been to use larger target volumes, while managing motion has led to the use of chest compression, breath-hold techniques and active gating of the treatment beam. Additionally, motion can be managed through the use of adaptive radiation therapy (ART) [13], with three forms in current use: offline, online and real-time. All operate by responding to anatomical changes with the difference being the time scale of the changes to which the methods are responding, as reviewed in [[14], [15], [16], [17], [18], [19]]. Offline ART reacts to changes over days or weeks (such as changes in tumour size), online ART reacts to day-to-day variations in anatomy and positioning (such as bladder filling or anatomical positioning), while real-time ART reacts to patient motion (such as breathing and bowel gas) that occur while the treatment is being delivered.

In 2016, an international multi-institutional study [20] was published documenting the dosimetric evaluation of four real-time adaptive radiotherapy systems across ten institutions. The study developed a unified testing framework with common protocols, motion traces and experimental procedures to evaluate the ability of the platforms to adapt in real-time. The study quantified the ability of all the tested platforms to improve the dosimetric accuracy of delivery to a moving target when real-time adaption is utilised compared to no motion correction. The study also provided a framework for testing new systems, for example the real-time adaptive capabilities of helical tomotherapy [[21], [22], [23]], released after the study was published.

There have been two commissioning and quality assurance (QA) papers published which focus on the adaptive capabilities of the helical tomotherapy platform. Both Goddard *et al.* [24] and AAPM Task Group 306 (TG306) [25] have recommended testing the real-time adaptive capabilities of the platform as part of routine QA. Where Goddard *et al.* demonstrated the tests they recommended, TG306 went into substantial detail regarding what was new with the system and provided comprehensive testing recommendations without providing details on how to perform the tests. The method utilised by Goddard *et al.* relied either on a modified patient specific quality assurance (PSQA) device or on a custom cradle, thus limiting the applicability of the method. Other publications have investigated the real-time adaptive capabilities of the helical tomotherapy platform [[26], [27], [28], [29]]. None of these papers demonstrated a methodology that can be reproduced to meet the requirements of either Goddard *et al.* or TG306.

In this paper we test the real-time adaptive capability of the helical tomotherapy platform across a series of patient derived lung and prostate traces using a methodology that can be replicated at any centre. Additionally, we demonstrate its use in a way that meets the requirements of Goddard *et al.* and TG306 and facilitates comparison against previously tested real-time adaptive technologies.

## 2 Materials and method

### 2.1 Treatment platform

The Radixact is a helical tomotherapy system. The Radixact Synchrony version includes kV imaging and optical surface tracking. The additional hardware, along with a software package, allows the Radixact Synchrony to perform real-time adaptive radiotherapy by modifying the treatment delivery according to the tracked target motion [21].

Treatment adaption is achieved in the superior/inferior direction via the jaws, used to define the superior and inferior radiation field, sliding back and forth on a track. In the left/right and anterior/posterior directions, the multi-leaf collimator (MLC) opening is shifted using a binary MLC.

### 2.2 Methodology and datasets

The unified testing framework developed for the international study [20] was used to quantify the delivery accuracy of Radixact Synchrony markerless and marker-based adaptive treatment approaches. The framework includes DICOM datasets, treatment plan protocols, lung and prostate motion traces (see Supplementary Figs. S1–S9) and experimental procedure (downloadable from GitHub [30]).

Anonymised lung and prostate DICOM CT and structure datasets were imported into the Accuray Precision treatment planning system. Treatment plans were generated using these datasets according to the prescription and dose constraints of the SBRT arm of Radiation Therapy Oncology Group (RTOG) 1021 for the lung plan [31] and the five-fraction arm of RTOG 0938 for the prostate plan [32].

### 2.3 Equipment

**Fig. 1:**
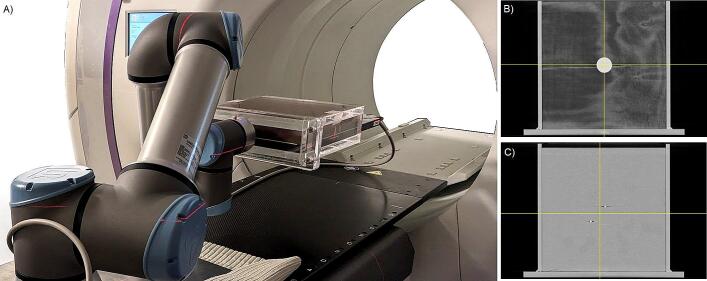
(a) Experimental setup of the robot actuating motion of the prostate phantom target and the 2D detector array dose measurement system on the Radixact couch. Coronal view from the CT of the phantoms showing the tracking object/s: (b) the 3D printed lung ‘target’ and (c) 2 of the 3 seeds in the prostate.

The motion platform was a small industrial robot, the UR16e (Universal Robots, Odense, Denmark) (Fig. 1*(a)*). An open-source software package (available on GitHub [33]) has been developed to allow the use of this robot as a 6DoF motion platform in radiation therapy [34] capable of manipulating a phantom up to 16 kg.

The detector used was the Octavius 1500 (PTW Dosimetry, Freiberg, Germany). To mount the detector to the motion platform, an acrylic box was designed to attach to the robot with one end open to allow the insertion of the detector and various build-up materials including different tracking targets. The box has 10 mm acrylic sides and back, and 25 mm acrylic top and bottom. A separate 10 mm piece of acrylic is used as a tool flange to facilitate mounting to and from the robot. The bespoke design is available on GitHub [33].

For the lung measurements, the Octavius 1500 was topped with 25 mm plywood and 5 mm solid water. In the centre of the plywood, a 38 mm diameter hole was drilled, into which a higher density cylinder mimicking a tumour was sandwiched between two thinner discs with densities similar to the plywood (Fig. 1*(b)*). All three discs were 3D printed, the ‘target’ disc was printed with PLA and the 2 plywood equivalent discs were printed with lightweight forming PLA [35].

For the prostate measurements, the Octavius 1500 was topped with 20 mm solid water, 10 mm slab bolus and 1 mm solid water (this 1 mm slab was to allow insertion and removal from the acrylic box). Three gold seeds were located on either side of the slab bolus to allow marker-based tracking of this setup (Fig. 1*(c)*).

### 2.4 Experimental procedure

Phantoms were scanned on a Siemens Somatom Confidence CT scanner (Siemens Healthineers, Erlangen, Germany) using a thorax protocol for the lung phantom and a pelvis protocol for the prostate phantom with 1 mm slice thickness and reconstructed using the Br38 kernel.

On the Radixact, the robot was mounted to a metal plate which was placed on the couch with a non-slip mat between the couch and baseplate (Fig. 1*(a)*). When using the lung phantom, the Synchrony external surface monitoring system tracked the phantom, meaning the system monitoring the 'external' motion of the phantom measured the same motion as the system monitoring the internal target.

The plans were delivered to the phantom while it was static, then with the phantom in motion, both with and without real-time adaption. Readout of the 2D array was performed using VeriSoft version 8.1.1.0 (PTW Dosimetry, Freiberg, Germany) and the dose was integrated over the total delivery. Analysis was also performed in VeriSoft, calculating the global γ for 1 %/1 mm, 2 %/2 mm and 3 %/3 mm pass criteria (relative to the maximum dose of the static reference with a 10 % low dose threshold).

## 3 Results

**Fig. 2:**
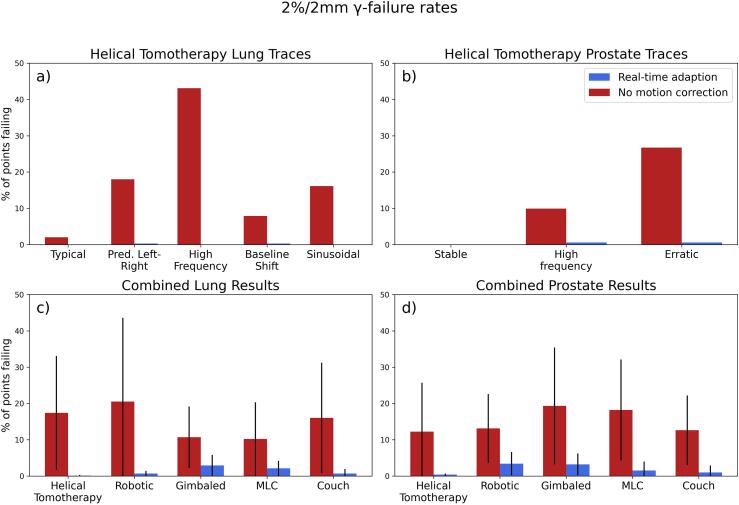
2 %/2 mm γ-fail rates for a) lung traces and b) prostate traces delivered on the helical tomotherapy system for motion adapted and no motion correction. Average 2 %/2 mm γ-fail rates across all c) lung and d) prostate traces (error bars are 1SD) for all real-time adaptive systems tested using the unified testing framework [20]. Note: helical tomotherapy prostate data only includes the three traces delivered successfully.

The γ-fail rate for the lung traces at 2 %/2 mm delivered using motion adaption was 0 %, 0.3 %, 0 %, 0.3 %, 0 % for the typical, predominantly left–right, high frequency, baseline shift and sinusoidal traces respectively. For no motion correction the γ-fail rate (2 %/2 mm) was 2.0 %, 18.0 %, 43.1 %, 7.9 % and 16.1 % for typical, predominantly left–right, high frequency, baseline shift and sinusoidal traces respectively (Fig. 2a).

The lung motion traces [36] vary in terms of amplitude, frequency and direction of motion. The motions with smaller amplitude (typical and baseline shift) appeared to have a lesser impact on delivery accuracy when no motion correction was utilised while those with higher amplitude (sinusoidal, predominantly left–right and high frequency) had substantially worse delivery accuracy when no motion correction was utilised.

The γ-fail rate for the prostate traces at 2 %/2 mm delivered using motion adaption was 0 %, 0.6 % and 0.6 % for the stable, high frequency and erratic traces respectively. For no motion correction, the γ-fail rate (2 %/2 mm) was 0 %, 9.9 % and 26.7 % for stable, high frequency and erratic traces respectively (Fig. 2b). For an example of the readout from the Octavius 1500, please see Supplementary Fig. S10, and for plots of the γ-fail results at other thresholds see Supplementary Figs. S11 and S12.

The prostate motion traces [37] vary in terms of amplitude and length of deviation and whether they return to baseline. All prostate motion traces were successfully adapted to by the helical thomotherapy system. Deliveries without motion correction demonstrated increased error when the length of deviation increased and when the motion didn’t return to baseline.

Fig. 2c compares the average γ-fail rate across all lung traces for the helical tomotherapy system against all the other systems tested using the unified testing framework. Fig. 2d compares the average γ-fail rate across all prostate traces for the helical tomotherapy system against all the other systems tested using the unified testing framework.

## 4 Discussion

The delivery accuracy of a helical tomotherapy system equipped with kV imaging and optical surface guidance for real-time adaptive radiotherapy was quantified using a unified testing framework previously published in an international study [20]. Performance of the helical tomotherapy system for both markerless and marker-based tracking was characterised and found to improve delivery accuracy for all tested motion traces.

TG306 recommends that the helical tomotherapy real-time adaptive system be evaluated monthly using an end-to-end test comparing an adaptive delivery to a detector in motion with the planned dose. The unified testing framework, as demonstrated in this work, would satisfy the requirements of TG306 if the plan was used instead of a static delivery. Although this work performed measurements in the coronal plane, the methodology could be adapted to also check agreement in the sagittal plane as required by TG306. The tolerances recommended by TG306 are appropriate when comparing against the planned dose in an end-to-end test. However, in situations where the aim is to evaluate the impact of motion on various treatment delivery methods, the recommendation from this work would be to use a static delivery as the reference. When evaluated in this way, a gamma pass rate of >95 % at 2 %/2 mm would be expected for real-time adapted deliveries regardless of method of adaption or the type of motion trace.

The helical tomotherapy results showed similar improvement in delivery accuracy compared with previously tested real-time adaptive modalities [20] (Fig. 2*c and d*). There was a range of dosimetry systems, motion platforms and degrees of freedom used in the international study, thus direct comparison is not possible, however, the method used in this experiment was consistent with the ethos of the international study which facilitated a comparison of outcomes using different motion adaption methodologies.

In summary, helical tomotherapy real-time adaption was shown to improve the accuracy of dose delivered to a moving phantom compared with no motion adaption. Real-time adaptive delivery using helical tomotherapy was found to be as effective at compensating for motion as robotic, gimbaled, multi-leaf collimator tracking and couch tracking real-time adaption methods.

## CRediT authorship contribution statement

**Jonathan Hindmarsh:** Conceptualization, Data curation, Formal analysis, Investigation, Methodology, Visualization, Writing – original draft. **Scott Crowe:** Formal analysis, Investigation, Methodology, Writing – review & editing. **Julia Johnson:** Resources, Writing – review & editing. **Chandrima Sengupta:** Software, Writing – review & editing. **Jemma Walsh:** Resources, Writing – review & editing. **Sonja Dieterich:** Conceptualization, Supervision, Writing – review & editing. **Jeremy Booth:** Conceptualization, Supervision, Writing – review & editing. **Paul Keall:** Conceptualization, Methodology, Supervision, Writing – review & editing.

## Funding

This research was supported by a Cancer Institute NSW Translational Program Grant (2019/TPG2165) and an 10.13039/100015539Australian Government Research Training Program (RTP) Scholarship. P Keall acknowledges funding from an Australian Government National Health and Medical Research Council (APP1194004) Investigator grant.

## Declaration of competing interest

The authors declare that they have no known competing financial interests or personal relationships that could have appeared to influence the work reported in this paper.
