# Supplementary material for: A dosimetric comparison of helical tomotherapy treatment delivery with real-time adaption and no motion correction
Source: Phys Imaging Radiat Oncol. 2025 Mar 5;34:100741. doi: 10.1016/j.phro.2025.100741 (PMC11931245; doi:10.1016/j.phro.2025.100741)
Supplement: Supplementary Data 1 [file mmc1.pdf]

**Supplementary materials:** The lung and prostate motion traces used to program the robot holding the measurement array for the dosimetric experiments.

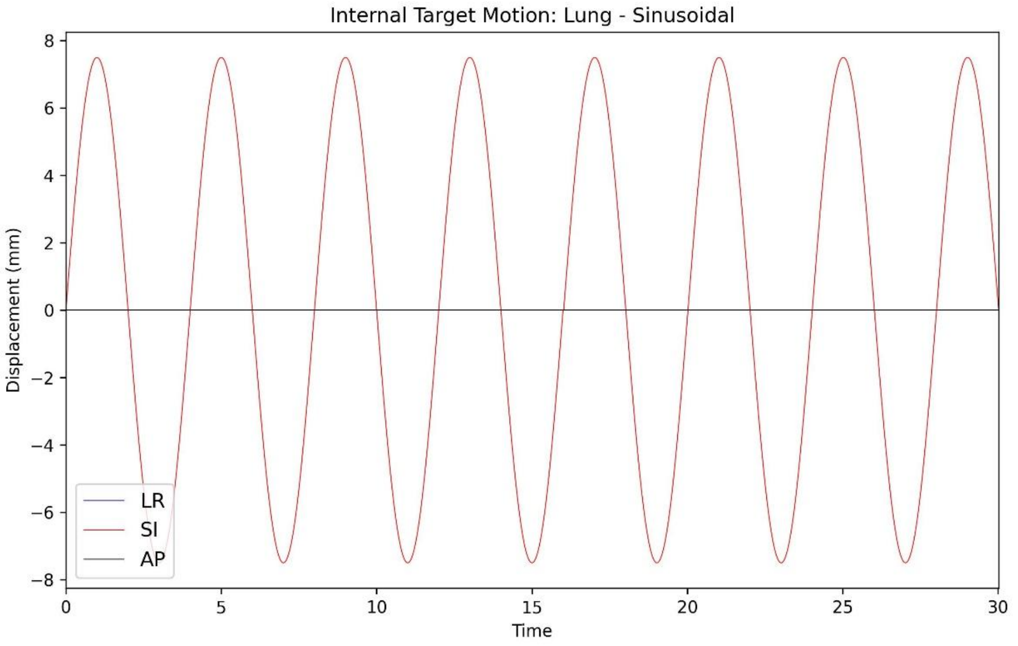


*Figure S1: Sinusoidal target motion (lung).*

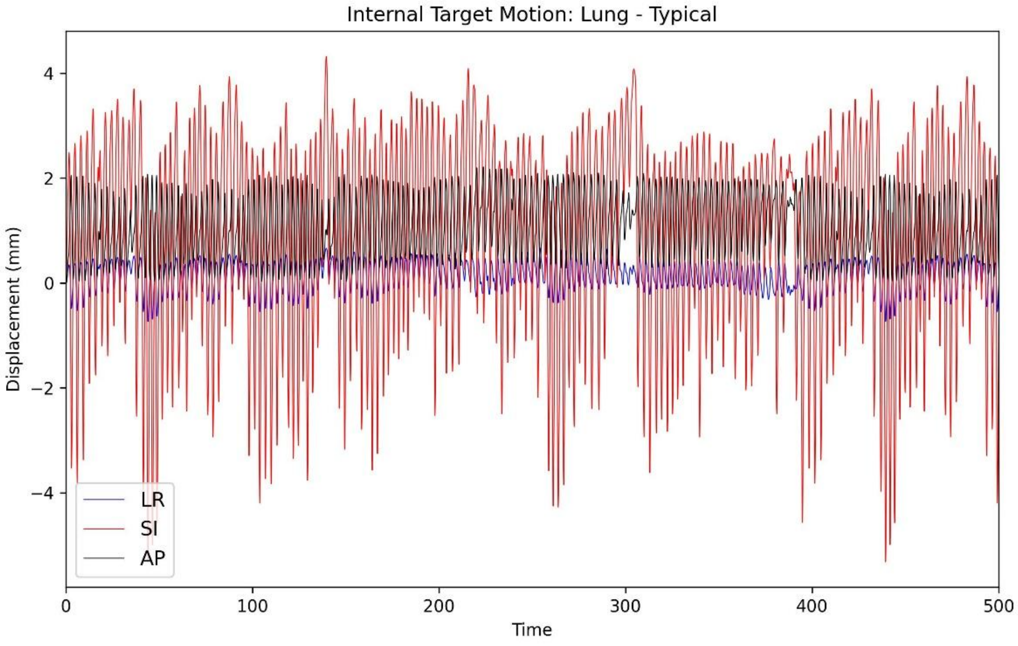


*Figure S2: Typical lung target motion (lung).*

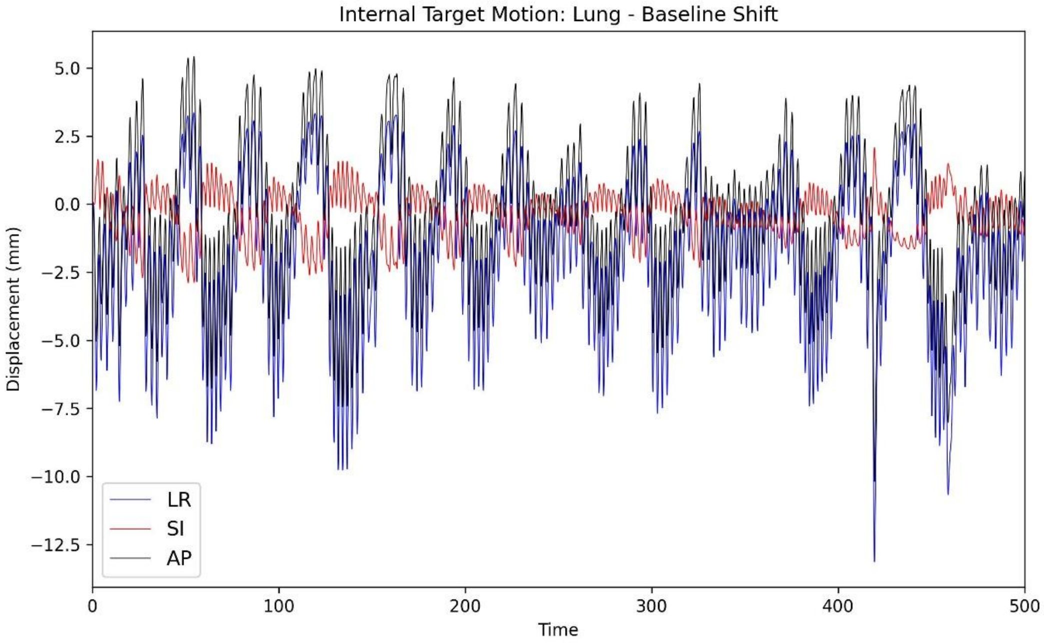


*Figure S3: Baseline shift target motion (lung).*

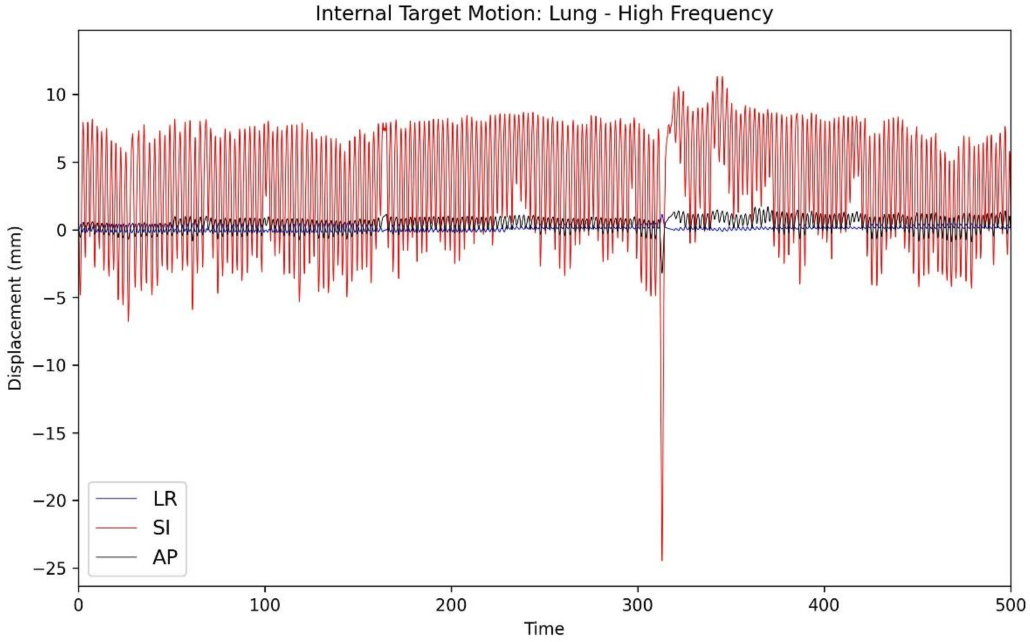


*Figure S4: High frequency target motion (lung).*

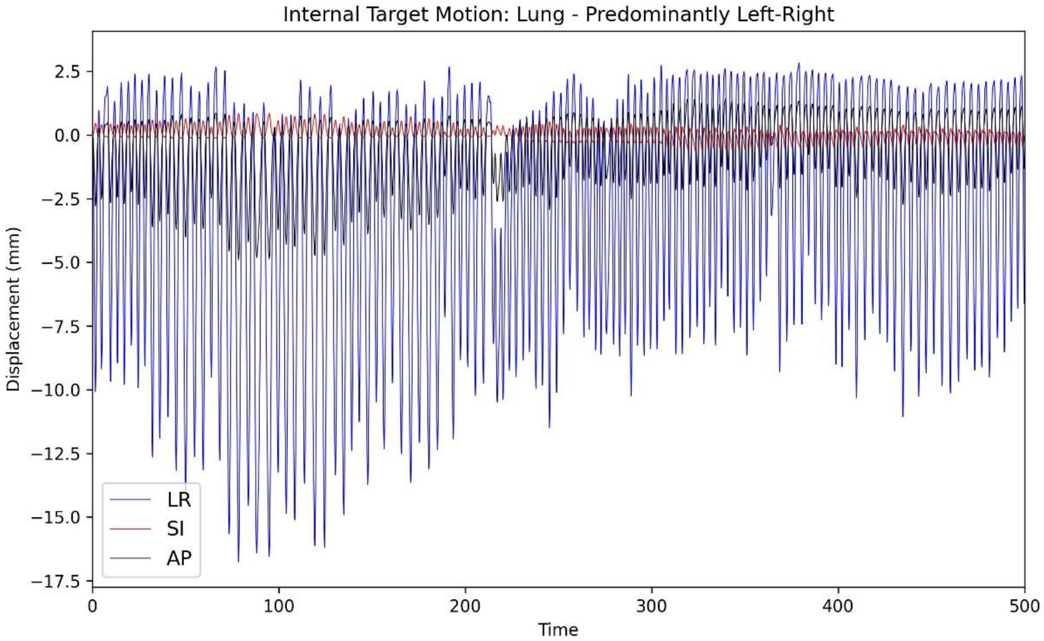


*Figure S5: Predominantly left-right target motion (lung).*

The original trajectories were estimated by a Cyberknife Synchrony system (Accuray Inc., Sunnyvale, CA) at Georgetown University Hospital, during stereotactic body radiotherapy.
Suh Y, Dieterich S, Cho B, Keall PJ. An analysis of thoracic and abdominal tumour motion for stereotactic body radiotherapy patients. Phys Med Biol 2008;53:3623. https://doi.org/10.1088/0031-9155/53/13/016.

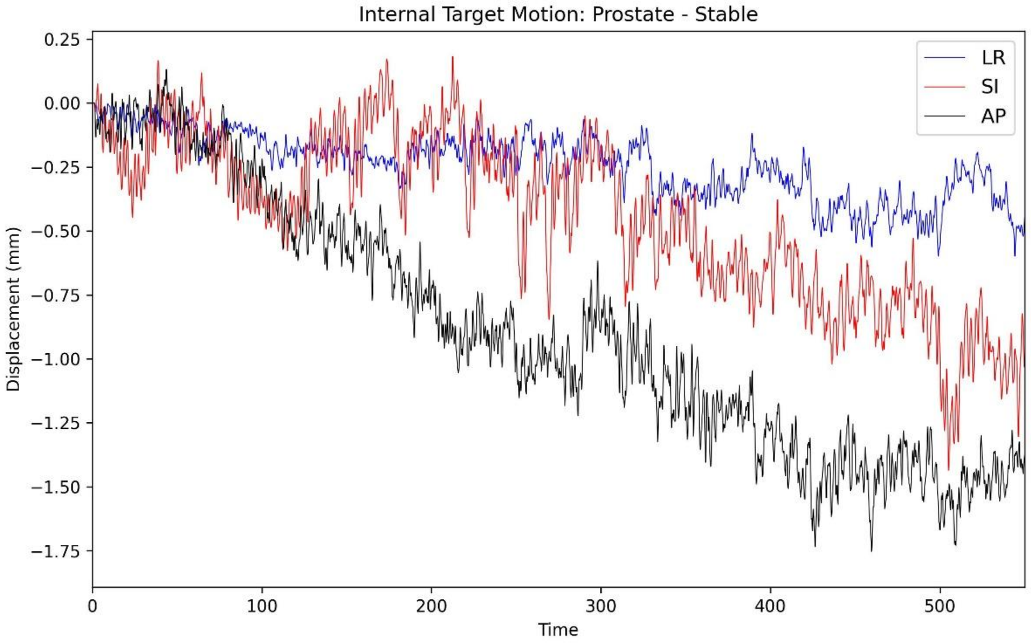


*Figure S6: Stable target motion (prostate).*

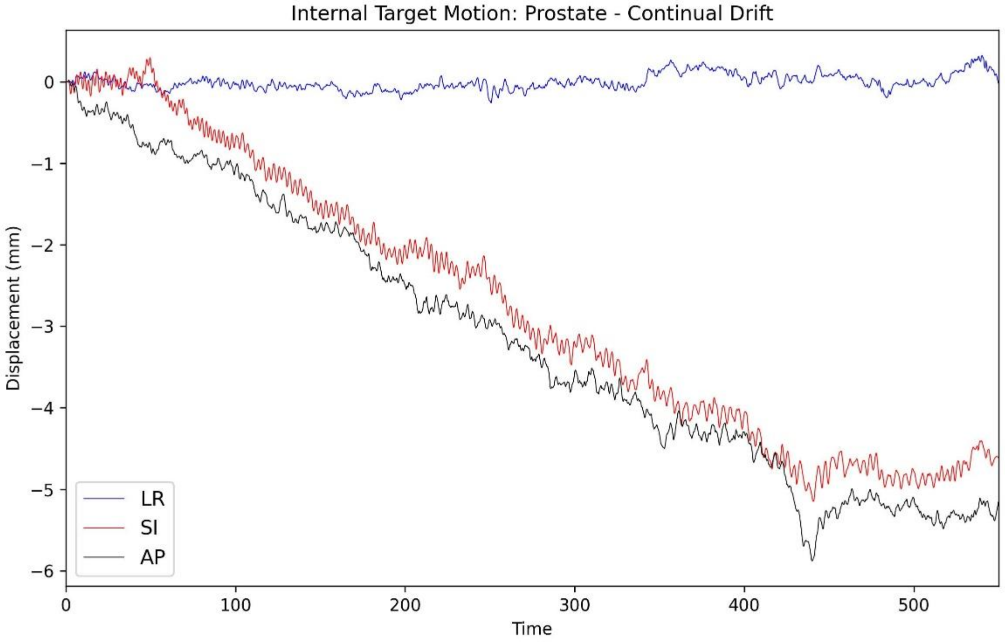


*Figure S7: Continual drift target motion (prostate).*

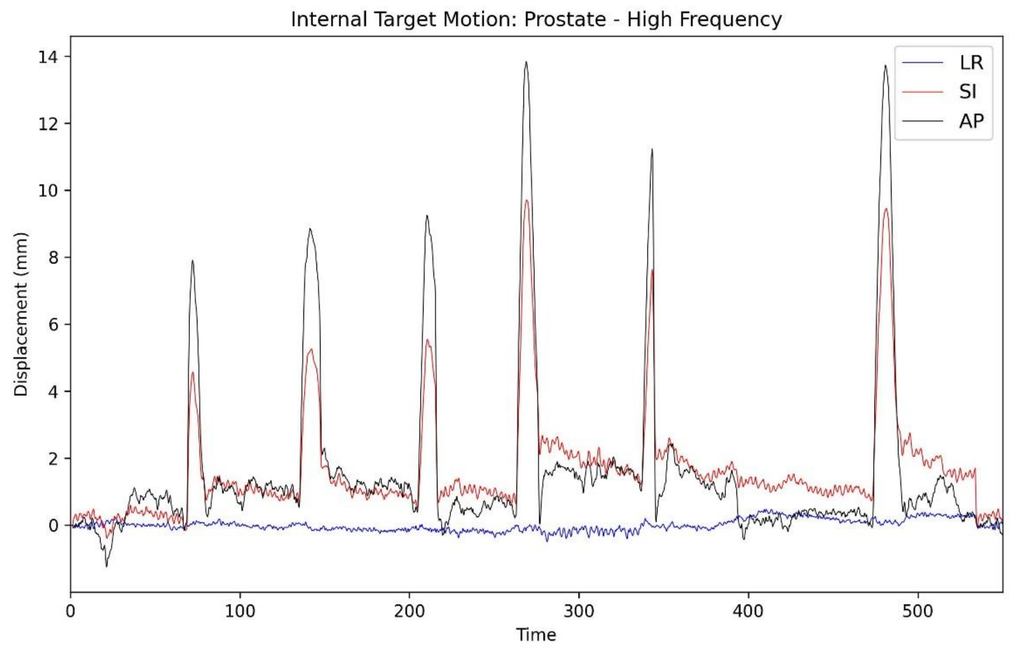


*Figure S8: High frequency target motion (prostate).*

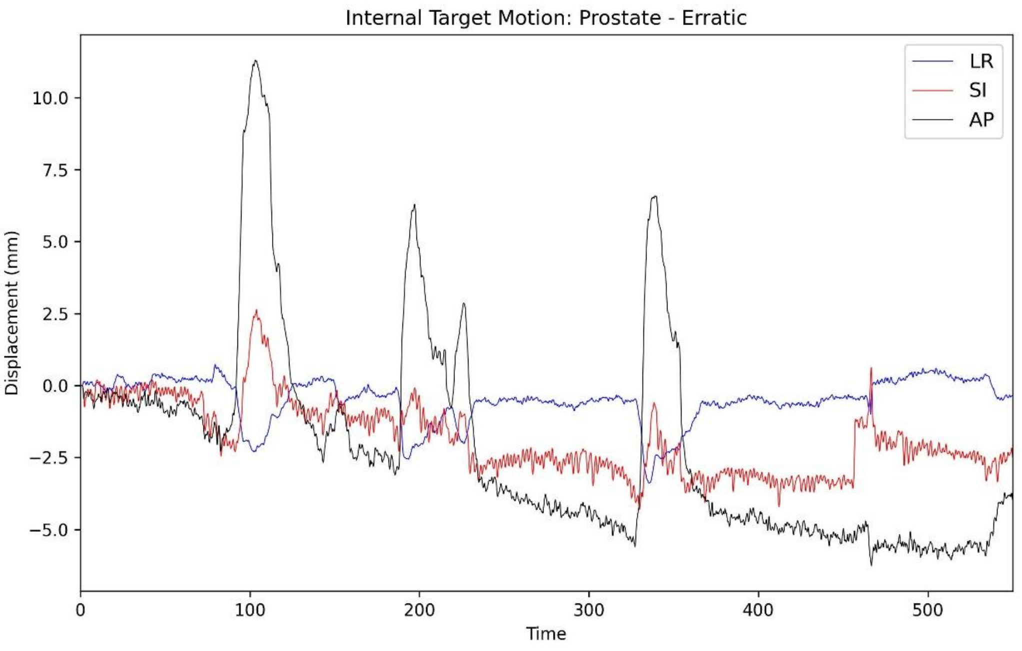


*Figure S9: Erratic target motion (prostate).*

Original trajectories were from Calypso data obtained during prostate radiotherapy treatments by Drs Katja Langen and Patrick Kupelian at MD Anderson Cancer Center Orlando.

Langen KM, Willoughby TR, Meeks SL, Santhanam A, Cunningham A, Levine L, et al. Observations on Real-Time Prostate Gland Motion Using Electromagnetic Tracking. International Journal of Radiation Oncology*Biology*Physics 2008;71:1084–90. https://doi.org/10.1016/j.ijrobp.2007.11.054.

*Table S1: Materials and methods for the measurement, including planning and delivery systems, experimental set ups and measurements obtained.*

| **Study** | **Institution** | **System version** | **Planning System** | **Treatment type** | **Motion guidance** | **Motion platform** | **Dosimetry phantom** | **Degrees of freedom** |
|---|---|---|---|---|---|---|---|---|
| This work | Radiation Oncology, Royal Brisbane Women's Hospital | Radixact Synchrony | Accuray Precision | TomoTherapy | kV and optical imaging | Custom | Custom phantom for Octavius 1500 | Lung: 3D<br>Prostate: 3D |
| **International Study** | 10 centres worldwide | CyberKnife, Vero, MLC tracking, Couch tracking | Multiplan, iPlan, Eclipse, Pinnacle | Robotic, IMRT, Conformal, VMAT | kV and optical, Calypso, Optical ExacTrac, Motion platform, Optical | Custom, CIRS dynamic phantom, QUASAR, BrainLab gating phantom, Hexamotion, Hexapod | Octavius, Delta4, ArcCHECK, Quasar respiratory phantom, I'mRT, Stereotactic dose verification phantom | Lung: 1D, 3D or 4D (3D target motion+ external surrogate)<br>Prostate: 1D, 2D, or 3D |

kV = kilovoltage
D = Degrees of freedom

*Table S2: Mean and range of the reported planning values and average delivery time for the lung and prostate plans compared to the range reported in the international study [17].*

| | Radixact Synchrony | Range from International Study |
|---|---|---|
| *Lung* | | |
| Conformity Index (CI) | 0.95 | (0.97-1.2) |
| Homogeneity Index (HI) | 0.06 | (0.17-0.68) |
| Mean lung dose (Gy) | 3.6 | (2.7-4.2) |
| Treatment time (min) | 9.3 | (6.2-43) |
| | | |
| *Prostate* | | |
| CI | 0.78 | (0.97-1.21) |
| HI | 0.06 | (0.02-0.17) |
| Mean rectal dose (Gy) | 8.63 | (7.89-16.93) |
| Mean bladder dose (Gy) | 6.88 | (5.9-14.3) |
| Treatment time (min) | 5.1 | (3-43.5) |

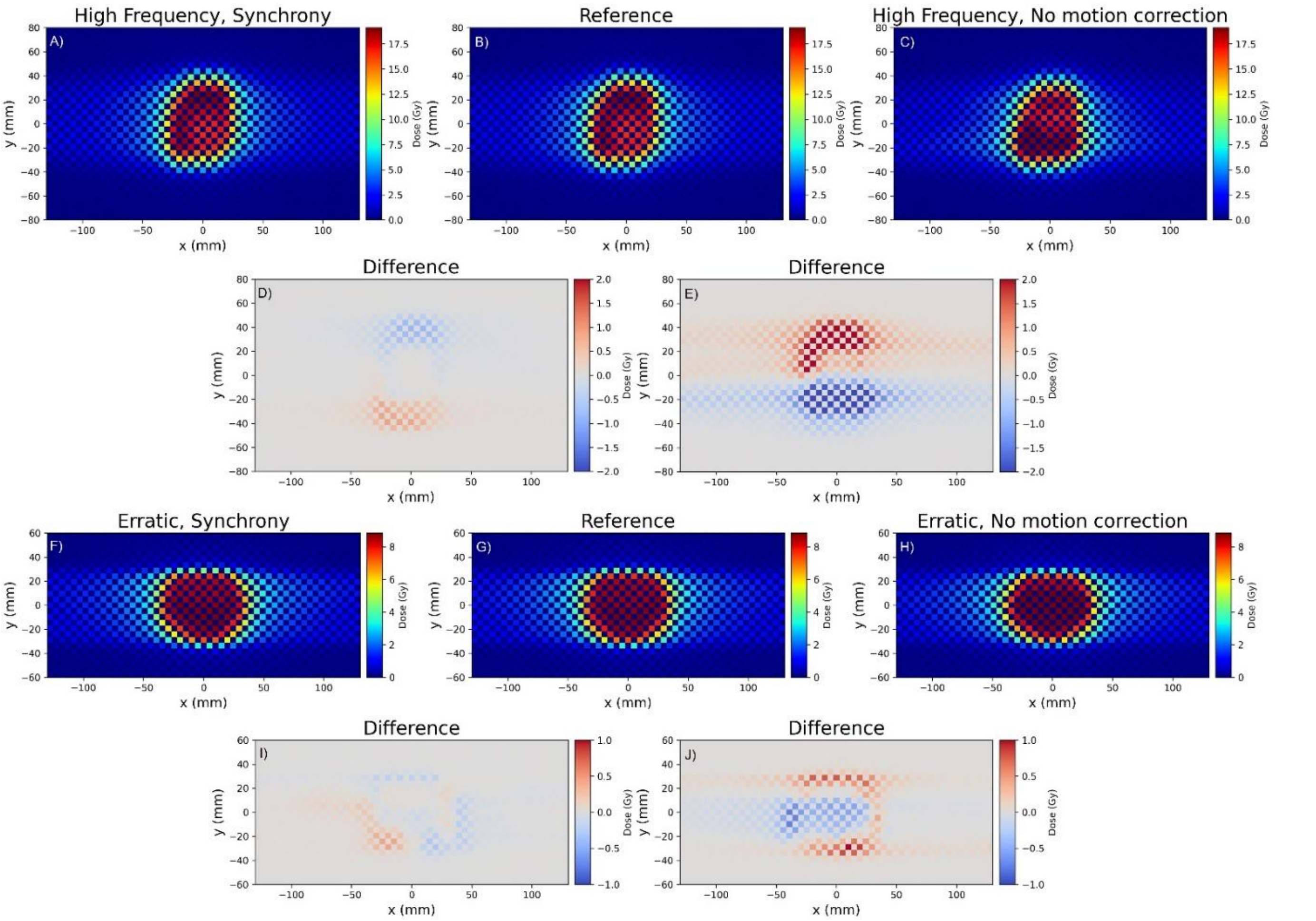


*Figure S10: Dose distributions from Octavius 1500 for lung (b) reference (static delivery), and lung High Frequency motion (a) Synchrony adapted and (c) without motion correction. Dose difference between reference and (d) Synchrony and (e) no motion correction. And prostate (g) reference (static delivery), and Erratic prostate motion (f) Synchrony adapted and (h) without motion correction. Dose difference between reference and (i) Synchrony and (j) no motion correction.*

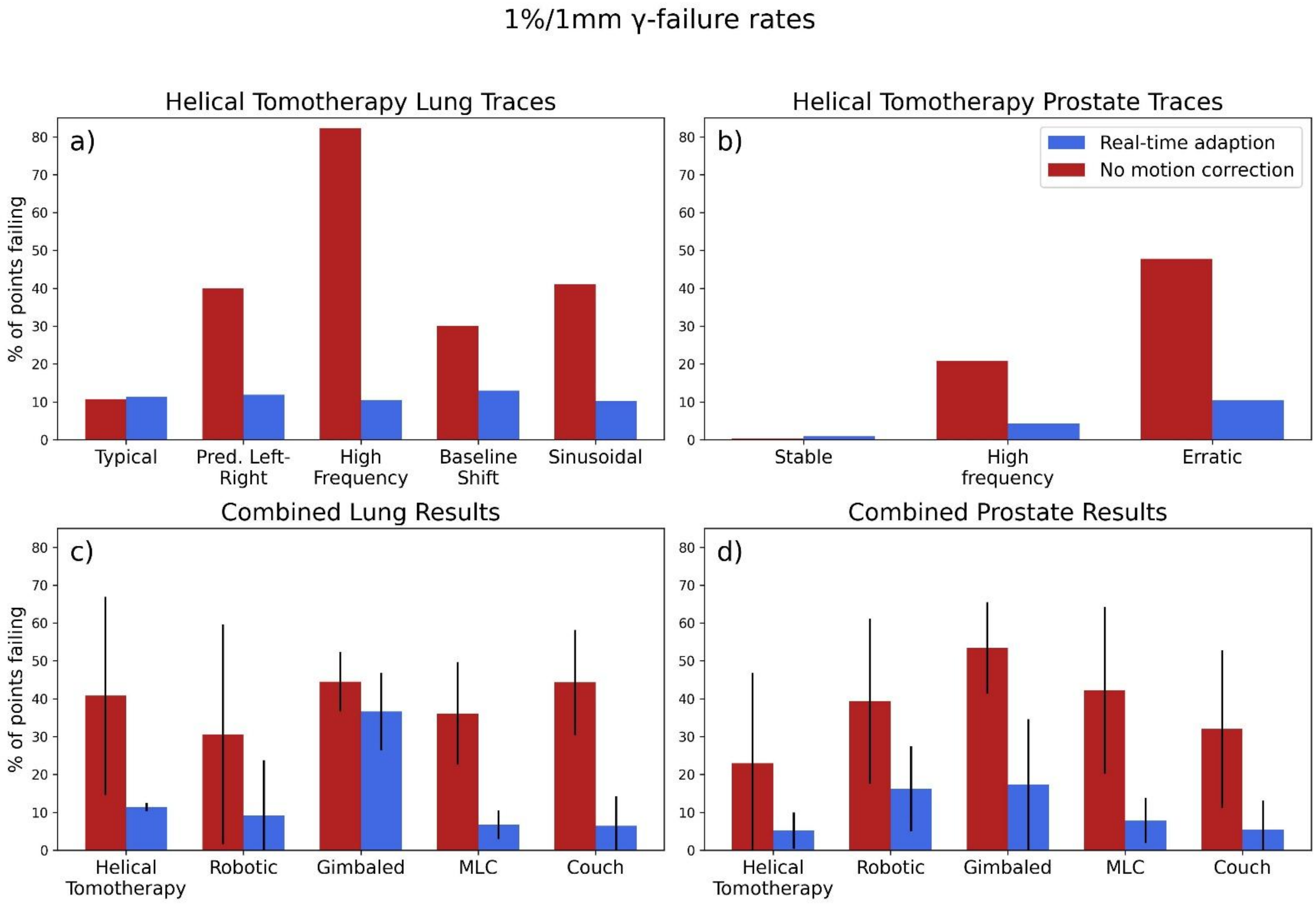


*Figure S11: 1%/1mm γ-fail rates for a) lung traces and b) prostate traces delivered on the Radixact for Synchrony adapted and no motion correction. Average 1%/1mm γ-fail rates across all c) lung and d) prostate traces (error bars are 1SD) for all real-time adaptive systems tested using the unified testing framework [20]. Note: Radixact prostate data only includes the three traces delivered successfully*

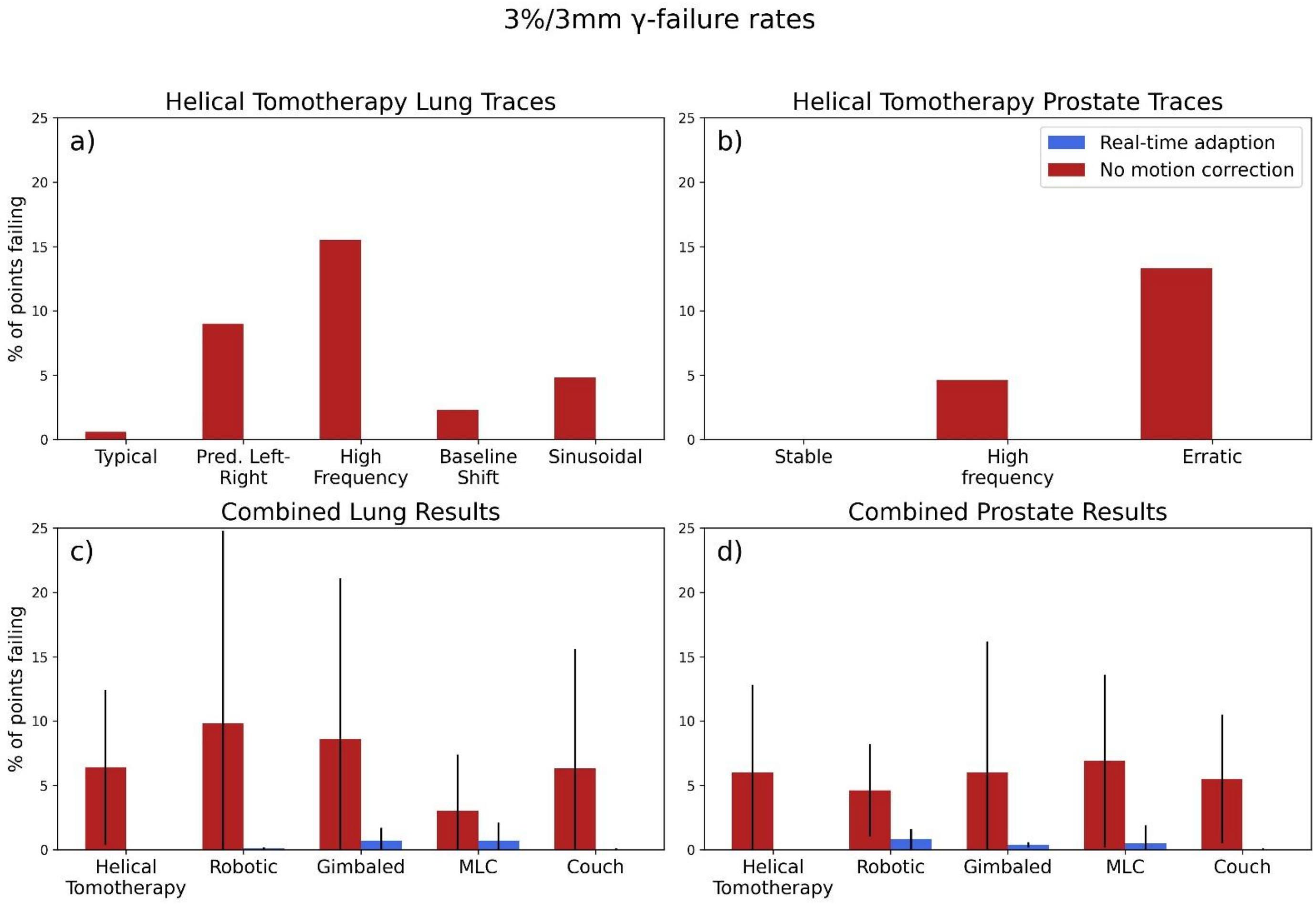


*Figure S12: 3%/3mm γ-fail rates for a) lung traces and b) prostate traces delivered on the Radixact for Synchrony adapted and no motion correction. Average 3%/3mm γ-fail rates across all c) lung and d) prostate traces (error bars are 1SD) for all real-time adaptive systems tested using the unified testing framework [20]. Note: Radixact prostate data only includes the three traces delivered successfully*
